# Effects of autoregulated and non-autoregulated blood flow restriction on vastus medialis oblique responses during low-load resistance exercise

**DOI:** 10.3389/fspor.2025.1712606

**Published:** 2025-12-12

**Authors:** Masoud Moghaddam, Michael C. Rabel, Tim Werner, Nicholas Rolnick

**Affiliations:** 1 Department of Physical Therapy, University of Maryland Eastern Shore, Princess Anne, MD, United States; 2 Department of Exercise Science, Salisbury University, Salisbury, MD, United States; 3 PHITWELL, New York, NY, United States; 4 The Human Performance Mechanic, Manhattan, NY, United States

**Keywords:** autoregulation, muscle hypertrophy, muscle thickness, near-infrared spectroscopy, perceptual responses

## Abstract

**Introduction/purpose:**

Blood flow restriction (BFR) training is a viable strategy for inducing muscle hypertrophy and strength gains using low external loads. This study investigated the acute effects of autoregulated BFR (AR-BFR) and non-autoregulated BFR (NAR-BFR) using the Delfi Personalized Tourniquet System, and no BFR on vastus medialis obliquus (VMO) thickness, muscle oxygenation, hemoglobin concentration, and perceptual responses [rating of perceived exertion (RPE) and rating of perceived discomfort (RPD)].

**Methods:**

Eighteen healthy adults (24.6 ± 2.3 years; 10 males, 8 females) performed a standardized single-leg wall squat protocol (1 × 30, 3 × 15 reps) under each condition in a randomized, crossover design.

**Results:**

Both BFR conditions (AR-BFR and NAR-BFR) resulted in significantly greater increases in VMO thickness compared to the no BFR condition (*p* < 0.05), with no difference between the two BFR modalities (*p* > 0.05). Oxygen saturation significantly decreased across sets in both BFR conditions relative to no BFR (*p* < 0.05). However, AR-BFR initially maintained higher oxygen levels before declining to values comparable to those of NAR-BFR. Total hemoglobin levels were significantly elevated in both BFR conditions compared to no BFR (*p* < 0.05), with NAR-BFR producing the highest levels during the early sets. Both RPE and RPD were significantly greater in AR-BFR and NAR-BFR compared to no BFR (*p* < 0.05), with no significant differences between the two BFR protocols (*p* > 0.05).

**Conclusion:**

These findings suggest that BFR training, regardless of autoregulated pressure application during exercise, produces acute physiological responses such as muscle swelling, localized **hypoxia**, and hemoconcentration that are conducive to initiating a stimulus that could elicit muscle hypertrophy in a training program. Although elevated perceptual demands accompanied these responses, the minimized mechanical joint stress supports the use of BFR as a clinically relevant alternative to high-load resistance training for individuals with limited tolerance to mechanical loading.

## Introduction

Blood flow restriction (BFR) training has gained popularity in both clinical rehabilitation and athletic performance settings due to its ability to promote muscle hypertrophy and strength at relatively low exercise intensities (1–4). This method is attractive because it minimizes joint stress while still producing adaptations comparable to high-load training, making it suitable for a wide range of populations. The BFR method applies a pneumatic cuff around the limb proximally to restrict venous return and partially occlude arterial blood flow during low-intensity exercise at 20%–40% of one-repetition maximum (1RM) to induce muscle growth (1, 5–7). This technique creates a significant hypoxic environment during exercise, increasing metabolic stress, fatigue, and cellular swelling (e.g., muscle thickness) in the working muscles (1–4).

Conventional BFR training typically uses static pressure settings that remain fixed during exercise. These fixed pressures do not account for changes in limb circumference during muscle activation, which may affect the applied stimulus (7–9). To address this limitation in BFR methodology, autoregulated BFR (AR-BFR) has been developed, where cuff pressure dynamically adjusts throughout the range of motion in response to muscle contraction (10–12). Although previous studies (10, 11, 13) have examined cardiovascular responses to AR-BFR and non-autoregulated BFR (NAR-BFR), no study to date has assessed acute morphological adaptations in the vastus medialis oblique using ultrasound. Our study also employed a non-failure exercise protocol, expanding on existing methodologies.

The vastus medialis oblique (VMO) represents the most distal fibers of the vastus medialis muscle and plays a crucial role in maintaining proper patellar tracking, preventing excessive lateral displacement of the patella, and contributing to knee joint stability (14–16). Despite its importance, no study has examined acute changes in VMO muscle thickness and hemodynamics under AR-BFR vs. NAR-BFR during non-failure exercise. Healthy participants provide an ideal model to test these questions prior to recruitment of clinical populations where VMO function may be altered (17, 18).

Therefore, the purpose of this study was to investigate the acute effects of VMO-targeted exercise, both with and without autoregulated BFR, as well as a no-BFR reference protocol, on VMO morphology, muscle oxygenation, total hemoglobin levels, rating of perceived exertion (RPE), and rating of perceived discomfort (RPD). We hypothesized that both BFR conditions would produce greater reductions in oxygen saturation, higher hemoglobin levels, and greater muscle swelling compared to the no BFR condition. Additionally, we expected AR-BFR to attenuate these responses relative to NAR-BFR, due to its dynamic pressure adjustments.

## Methodology

### Experimental design

The proposed study employed a crossover, randomized-controlled, within-subjects design to investigate the acute physiological responses and muscle morphology resulting from implementing AR-BFR, NAR-BFR, and no BFR conditions during exercise. Each participant attended the laboratory on three separate occasions. During the initial session, participants provided informed consent, underwent a comprehensive health screening [including resting blood pressure and the Physical Activity Readiness Questionnaire (PAR-Q)], and drew a number to determine which condition (AR-BFR, NAR-BFR, or no BFR) they would receive on the first day. Subsequently, height, weight, and age were obtained, and participants kicked a soccer ball into a net to determine leg dominance. Once the dominant leg was determined, pre-exercise VMO thickness was assessed via ultrasound. Participants then familiarized themselves with the exercise protocol and set their proper landmarks for 40–45 degrees of knee flexion during the single-leg wall squat. Once the range was marked on the floor with adhesive tape, the near-infrared spectroscopy sensor was placed on the VMO to monitor and measure relevant physiological changes. Participants then performed the four-set exercise protocol. The RPE and RPD levels (0–10) were recorded after each set during every session. Immediately after the protocol, post-exercise VMO thickness was measured using ultrasound. The procedure was repeated during the second and third visits without repeating the initial health screening, height measurements, and consent process. There was a one-week gap between each session to minimize any potential effects on exercise performance and recovery. Each session was scheduled at approximately the same time during the day (±1 h) to control for diurnal variations in physiological responses.

### Participants

A total of eighteen adults (24.6 ± 2.3 years; 10 males, 8 females) were recruited for the study (see Table 1). Participants were classified as healthy based on ACSM guidelines (19) and a health screening that confirmed the absence of cardiovascular, metabolic, or musculoskeletal conditions. Resting systolic blood pressure values, though elevated in some individuals, did not exceed 140 mmHg, and no participants reported a diagnosis of hypertension. Participants had consistently engaged in an exercise program for at least 30 min, three times per week, over the past six months. They demonstrated their ability to perform a single-leg wall squat during the familiarization session. Before enrollment, all participants underwent a thorough health screening process to ensure they were in good health, free from any signs or symptoms of disease, and without any musculoskeletal injuries within the previous six months. Throughout the study duration, participants were instructed to maintain their regular training activities while abstaining from exercise-related activities, caffeine, and alcohol for 24 h prior to each session. Each participant signed an informed consent document in accordance with the Declaration of Helsinki, acknowledging potential risks and harm. The study was approved by the Institutional Review Board of the University of Maryland Eastern Shore (protocol #01-2024-006).

**Table 1:** Baseline participant characteristics.

Sex	Males	Females	Combined
Sample size	*n* = 10	*n* = 8	*N* = 18
Age (years)	24.50 ± 1.35	24.75 ± 3.24	24.61 ± 2.30
Height (cm)	183.47 ± 7.62	166.19 ± 8.53	175.79 ± 11.78
Body mass (kg)	90.93 ± 10.10	71.49 ± 15.03	82.29 ± 15.68
BMI (kg/m^2^)	27.11 ± 3.58	25.85 ± 5.28	26.55 ± 4.32
SBP (mmHg)	133 ± 7	115 ± 10	125 ± 12
DBP (mmHg)	78 ± 8	82 ± 9	80 ± 8

cm, centimeters; kg, kilograms; BMI, body mass index; kg/m^2^, kilograms per meters squared; SBP, systolic blood pressure; DBP, diastolic blood pressure; mmHg, millimeters of mercury; α = 0.05; values expressed as mean ± SD.

### Vastus medialis oblique (VMO) thickness

The thickness of the VMO was assessed using a brightness-mode (B-mode) ultrasound imaging system (Biosound Esaote MyLab Gold, Universal Diagnostic Solutions, Inc., Vista, CA, USA) equipped with a multi-frequency linear array probe. The measurement site for VMO thickness was marked at 85% of the distance between the anterior superior iliac spine (ASIS) and the superomedial edge of the patella. The marking was made using indelible ink to ensure proper alignment and reproducibility during each scan before and after exercise. Before each scan, water-soluble gel was carefully applied to the skin and probe to enhance acoustic coupling. All ultrasound scans were analyzed using ImageJ software (version 1.53t) provided by the National Institutes of Health (NIH). The VMO thickness was determined by carefully identifying and measuring the distance between the superficial and deep aponeuroses around the muscle. The superficial aponeurosis is the outermost connective tissue layer, and the deep aponeurosis lies beneath the muscle belly. Accurate measurements were taken by positioning the ImageJ straight line tool's crosshair at the junction of the muscle and aponeurosis, following the muscle's contour for precise and reproducible thickness readings. All ultrasound scans were conducted by the same experienced researcher with an appropriate intra-class correlation coefficient (ICC) of 0.99 and a standard error of measurement (SEM) of 0.79 mm for VMO thickness.

### Vastus medialis oblique oxygenation measures

During the exercise protocol, a near-infrared spectroscopy (NIRS) sensor (MOXY muscle monitor, Fortiori Design LLC, Hutchinson, MN, USA) was placed on the participants' VMO to assess two primary hemodynamic variables: muscle oxygen saturation and total hemoglobin concentration (THb). The NIRS sensor was placed on the skin surface over the VMO at approximately 85% of the distance between the ASIS and the superomedial border of the patella. This site was selected just medially adjacent to the straight line between the ASIS and the superomedial border of the patella on the dominant leg. The sensor was secured with a medical-grade adhesive tape to ensure proper sensor contact and reduce the potential for movement artifacts. The NIRS sensor software continuously recorded oxygen saturation and total hemoglobin levels of the VMO during the exercise. The beginning and end of each exercise set were marked using the software's “mark function” to track the relevant time points. The average values for oxygen saturation and total hemoglobin for each set were calculated and used to analyze local muscle oxygenation and hemoconcentration responses throughout the exercise protocol.

### Rating of perceived exertion and discomfort

The participants' perceived exertion was assessed using the Borg 0–10 RPE scale, which was recorded at the end of each set to measure the subjective intensity of the exercise. After each set, participants were asked to verbally indicate their perceived level of effort, ranging from 0 (no exertion at all) to 10 (maximal exertion). The same researcher collected the RPE scores to ensure accurate reporting. Perceived discomfort was assessed using a 0–10 scale, which was recorded at the end of each set to evaluate participants' subjective discomfort during that set. After completing each set, participants were asked, “How much discomfort do you feel?” to indicate their perceived level of discomfort, with responses ranging from 0 (no discomfort) to 10 (maximal discomfort). To ensure consistency and accurate reporting, the same researcher collected the RPD data for all participants. Both RPE and RPD measurements were conducted in accordance with a previous validation study (20).

### Exercise protocol

**Figure 1:**
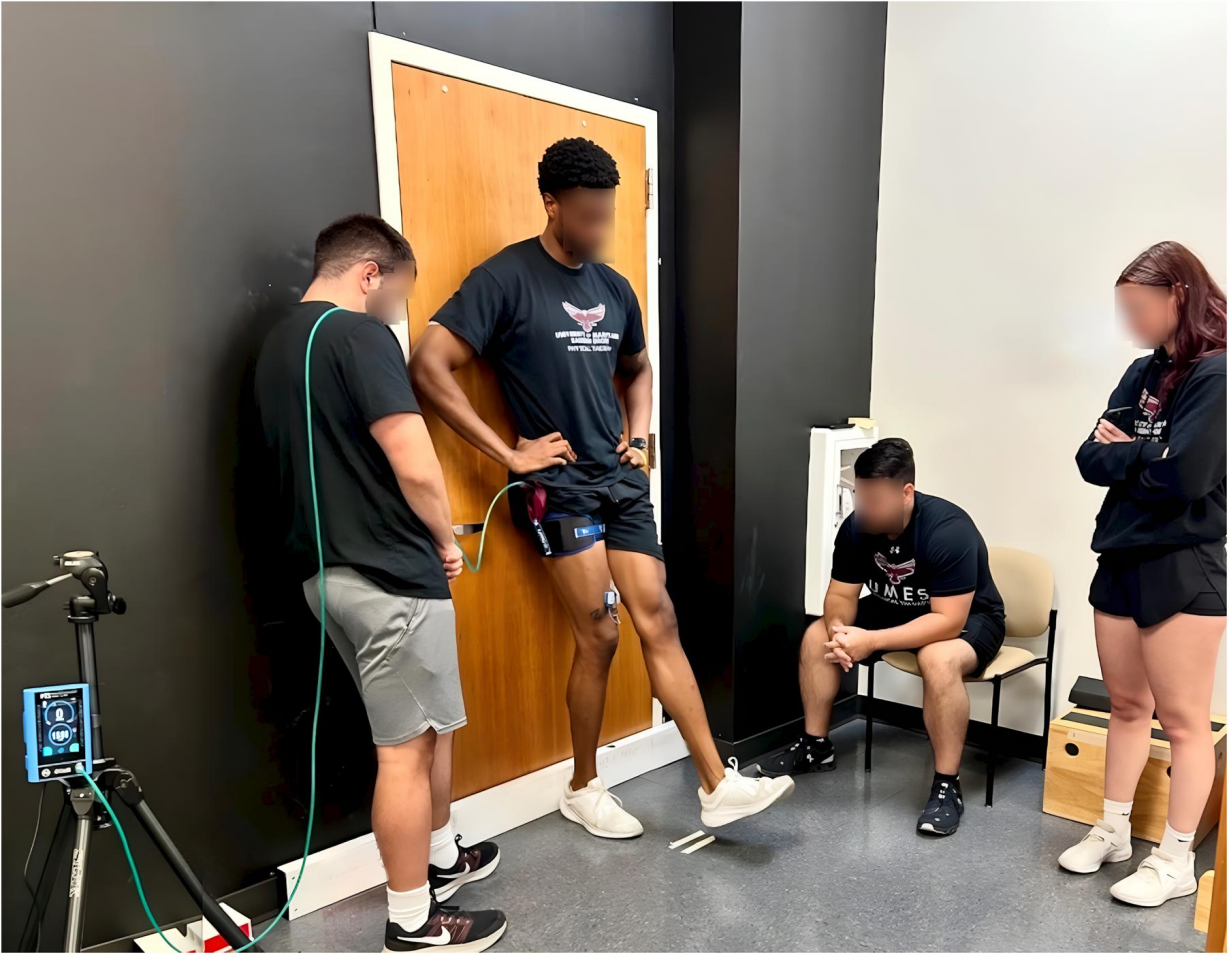
Single-leg wall squat protocol was used for all experimental conditions. Participants performed 75 total repetitions using a 30/15/15/15 protocol. Squat depth was standardized to 40–45° of knee flexion using floor markers aligned with the contralateral heel. Repetitions were paced by a metronome at a 3-second cadence (1.5 s eccentric and 1.5 s concentric). A 30-second rest interval was provided between sets. This protocol was designed to target the vastus medialis oblique (VMO) under consistent load and range of motion.

Participants performed a bodyweight single-leg wall squat exercise using a standard fixed repetition (1 × 30, 3 × 15) BFR protocol for a total of 75 repetitions (6, 7). Bodyweight was measured before each session to ensure there were no significant changes in external load. Exercise intensity was affected by the external load (bodyweight), the exercise task performed (squat depth), and fatigue accumulation (BFR-induced metabolic stress). To ensure consistency in the depth of the squat, the knee flexion range of motion was measured before the exercise began, with the desired range set between 40° and 45° of knee flexion. To facilitate this, the heel strike of the contralateral limb was matched with a piece of tape placed on the floor at both the 40- and 45-degree positions (see Figure 1). They served as visual markers to guide the participant's squat depth. Participants were instructed to perform the squat protocol in sync with a metronome, ensuring a consistent pace throughout all repetitions. Each repetition was executed with a controlled cadence of 3 s for both the concentric and eccentric phases (1.5 s each). The protocol began with an initial set of 30 repetitions, followed by a brief 30-s rest period. The subsequent three sets consisted of 15 repetitions each, with participants maintaining the same cadence. A 30-s rest was provided between sets with the BFR continually applied. This exercise targeted the VMO to ensure consistent muscle activation and effort throughout the session (21).

### Blood flow restriction protocol

During the BFR sessions, an 11.5 cm-wide pneumatic cuff was applied by the same researcher around the upper part of each participant's dominant thigh. Before exercise, participants rested in a supine position for 10 min to ensure baseline conditions were established. Relevant BFR methodological considerations are described in Table 2 in accordance with recent reporting guidelines published to enhance methodological clarity (22). Limb occlusion pressure (LOP) was determined with participants in a supine position using the Delfi device's built-in algorithm, which detects the minimum pressure required to occlude arterial pulsations via pressure oscillations. The cuff pressure was then set at 60% of the LOP in the BFR conditions for the entire exercise session (2, 7, 8, 22) (see Table 2). To maintain uniformity, all participants used a 34-inch cuff. We used the Delfi Personalized Tourniquet device (Delfi, Vancouver, Canada) for the BFR intervention, which has been validated for its accuracy when compared to Doppler ultrasound measurements (23) and has been shown to maintain set/interface pressure throughout exercise and during the rest periods (24). Blood flow was consistently restricted during the exercise, with cuff inflation starting before the first set and deflation occurring after the last set was completed (approximately 5 min and 30 s). In the AR-BFR condition, the Delfi Personalized Tourniquet System automatically adjusted the pressure in response to changes in muscle volume during contractions. In contrast, for the NAR-BFR condition, the cuff pressure remained constant during exercise with a non-commercially available manufacturer setting that turns off autoregulation. Participants were blinded to the autoregulation for all trials to avoid potential bias.

**Table 2:** Methodological guidelines (22).

Category	
Tourniquet cuff apparatus properties	Reporting element
Manufacturer and model	Delfi Personalized Tourniquet Device
Bladder width	11.5 cm (4.5 inches)
Material	Nylon
Type of bladder system	Single-chambered
Shape	Easi-Fit Variable Contoured
Bladder length	Cuff circumferentially enclosed all participants limbs with bladder applied throughout on the limb (34-inch cuff)
BFR instrument apparatus capabilities	
Manufacturer and model	Delfi Personalized Tourniquet Device
Method of pressure measurement	Automatic via the device
Pressure regulation	Autoregulation and fixed-pressure (depending on randomized leg)
Validity & reliability of limb occlusion pressure measurement	Validated (23)
BFR pressure prescription	
Limb occlusion pressure	60%
Posture used for measurement of limb occlusion pressure	Supine
Timings of pressure application	Applied before each exercise including the rest periods and deflated immediately after
Target vs actual pressure applied	Reported by Hughes and colleagues (24)

### Statistical analysis

Normality and sphericity assumptions for repeated measures analysis of variance (ANOVA) were evaluated using Shapiro–Wilk's test and Mauchly's test prior to data analysis. To examine mean differences in pre- and post-testing across the three conditions (AR-BFR, NAR-BFR, and no BFR), two-way repeated measures ANOVAs were conducted for each variable. For cases involving significant interactions, follow-up analyses were conducted using one-way ANOVAs with Bonferroni corrections. If no significant interaction was detected, the main effects were then analyzed. All statistical analyses were performed using IBM SPSS Statistics software (Version 28.0, IBM Corp., Chicago, Illinois). The alpha (*α*) level was set at 0.05, and the results were reported as mean ± standard deviation (SD). G*Power 3.1.9.7 software (Kiel University, Germany) estimated a sample size of 18 participants for repeated measures two-way ANOVAs at *α* = 0.05 and *β* = 0.80 to detect an effect size of 0.33 (25). Effect sizes are reported as eta squared (*η_p_*^2^) and defined as: 0.01, small effect; 0.06, moderate effect; and ≥0.14, large effect (26).

## Results

The results of the Shapiro–Wilk test indicated that the assumption of normality was met for all variables (*p* > 0.05). Additionally, Mauchly's test revealed that the assumption of sphericity was not violated (*p* > 0.05), suggesting that the data met the necessary assumptions for repeated measures analysis.

### Exercise protocol

All participants completed the standard fixed repetition (1 × 30, 3 × 15) BFR protocol with a total of 75 repetitions of the bodyweight single-leg wall squat exercise across all conditions (AR-BFR, NAR-BFR, and no BFR; see Figure 1). No adverse events were recorded in any group.

### Vastus medialis oblique thickness

**Figure 2:**
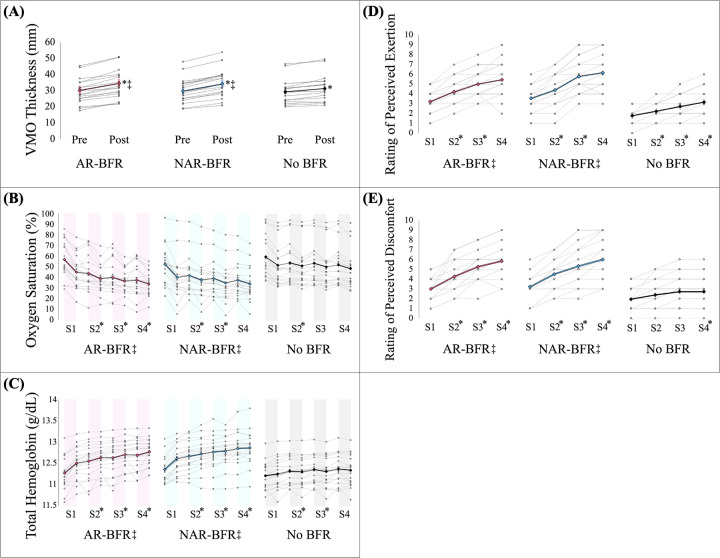
Acute exercise responses of the vastus medialis oblique (VMO) across three conditions: autoregulated (AR-BFR), non-autoregulated (NAR-BFR), and no BFR. Symbols: Significant time effect is indicated by * and significant interaction with no BFR by ‡. **(A)** VMO muscle thickness: Both AR-BFR and NAR-BFR resulted in significantly greater increases in VMO thickness compared to no BFR (*p* < 0.05), with no significant differences between AR-BFR and NAR-BFR (*p* > 0.05). **(B)** VMO muscle oxygen saturation: Both AR-BFR and NAR-BFR caused greater reductions in oxygen saturation from the second set onward compared to no BFR (*p* < 0.05). No significant differences were observed between AR-BFR and NAR-BFR (*p* > 0.05). **(C)** VMO total hemoglobin: NAR-BFR showed significantly higher total hemoglobin levels than AR-BFR and no BFR after the first set (*p* < 0.05). From the second set onward, both BFR conditions resulted in higher total hemoglobin compared to no BFR (*p* < 0.05), with no significant differences between BFR conditions (*p* > 0.05). **(D)** Rate of Perceived Exertion (RPE): Both BFR conditions resulted in significantly higher RPE compared to no BFR after each set (*p* < 0.05), with no significant differences between AR-BFR and NAR-BFR (*p* > 0.05). **(E)** Rate of Perceived Discomfort (RPD): Both BFR conditions elicited significantly higher discomfort than no BFR at all sets (*p* < 0.05), with no significant differences between AR-BFR and NAR-BFR (*p* > 0.05).

Our results revealed a significant time × condition interaction for VMO thickness, *F*(1,17) = 22.333, *p* < 0.05, *η_p_*^2^ = 0.568. Follow-up analyses indicated that all conditions (AR-BFR, NAR-BFR, and no BFR) significantly increased VMO thickness (*p* < 0.05). Specifically, both AR-BFR {12.48 ± 4.45% [95% confidence interval (CI): 10.27%–14.69%]} and NAR-BFR [13.05 ± 3.60% (95% CI: 11.26%–14.84%)] showed significantly greater increases in VMO thickness compared to the no BFR condition [5.95 ± 3.75% (95% CI: 4.09%–7.82%)] (*p* < 0.05), while there was no significant difference between the BFR conditions (*p* > 0.05) (see Table 3 and Figure 2A).

**Table 3:** Vastus Medialis oblique thickness (mm).

Condition	Pre	Post	*Δ*%
AR-BFR^‡^	29.12 ± 7.94	33.23 ± 8.83*	12.48 ± 4.45%
NAR-BFR^‡^	29.63 ± 7.96	34.03 ± 8.74*	13.05 ± 3.60%
No BFR	29.27 ± 7.60	31.22 ± 8.31*	5.95 ± 3.75%

BFR, blood flow restriction; AR-BFR, autoregulated BFR; NAR-BFR, non-autoregulated BFR; *Δ*%, percentage change; mm, millimeters.

* Significant time effect.

‡ Significant interaction with no BFR; α = 0.05; values expressed as mean ± SD.

### Vastus medialis oblique oxygen saturation

Our results showed a significant time × condition interaction for VMO oxygen saturation, *F*(1,17) = 8.398, *p* < 0.05, *η_p_*^2^ = 0.331. Follow-up analyses comparing oxygen saturation between conditions during each set revealed a significant difference between NAR-BFR and no BFR after the first set (*p* < 0.05), but no significant differences were observed between AR-BFR and no BFR, nor between the two BFR conditions (*p* > 0.05). During the second, third, and fourth sets, significant differences were found between both BFR conditions (AR-BFR and NAR-BFR) and no BFR (*p* < 0.05), while no significant differences were observed between the two BFR conditions (*p* > 0.05). Overall, no BFR resulted in the highest oxygen saturation levels throughout the exercise session. In the BFR conditions, AR-BFR showed higher oxygen saturation levels during the first and second sets compared to NAR-BFR. However, this pattern reversed during the third and fourth sets, where AR-BFR induced the lowest oxygen saturation levels compared to NAR-BFR (see Table 4 and Figure 2B).

**Table 4:** Average oxygen saturation during each Set (%).

Condition	Set 1	Set 2	Set 3	Set 4
AR-BFR^‡^	50.26 ± 15.20	40.88 ± 14.21*	37.89 ± 13.13*	35.35 ± 12.59*
NAR-BFR^‡^	46.35 ± 18.83	40.55 ± 18.41*	38.05 ± 16.40*	36.30 ± 15.29
No BFR	55.71 ± 18.91	52.84 ± 19.90*	51.46 ± 19.79	50.45 ± 19.23

BFR, blood flow restriction; AR-BFR, autoregulated BFR; NAR-BFR, non-autoregulated BFR; %, percentage.

* Significant time effect.

‡ Significant interaction with no BFR; α = 0.05; values expressed as mean ± SD.

Regarding time effects, oxygen saturation significantly decreased from the first to the second set [−26.08 ± 21.39% (95% CI: −36.72% to −15.45%)], from the second to the third set [−9.15 ± 15.83% (95% CI: −17.02% to −1.28%)], and from the third to the fourth set [−8.68 ± 11.37% (95% CI: −14.34% to −3.03%)] for AR-BFR (*p* < 0.05). For NAR-BFR, VMO oxygen saturation significantly decreased from the first to the second set [−15.85 ± 14.67% (95% CI: −23.14% to −8.55%)] and from the second to the third set [−5.72 ± 8.16% (95% CI: −9.78% to −1.67%)] (*p* < 0.05), but there was no significant change from the third to the fourth set [−5.11 ± 8.13% (95% CI: −9.16% to −1.07%)] (*p* > 0.05). In the no BFR condition, VMO oxygen saturation significantly decreased from the first to the second set [−6.67 ± 8.90% (95% CI: −11.10% to −2.24%)] (*p* < 0.05), but did not significantly change from the second to the third set [−2.66 ± 4.93% (95% CI: −5.11% to −0.21%)] and from the third to the fourth set [−2.02 ± 3.53% (95% CI: −3.78% to −0.26%)] (*p* > 0.05).

### Vastus medialis oblique total hemoglobin

Our results showed a significant time × condition interaction for VMO total hemoglobin levels, *F*(1,17) = 10.617, *p* < 0.05, *η_p_*^2^ = 0.384. Follow-up analyses comparing total hemoglobin levels between conditions during each set revealed that after the first set, total hemoglobin levels were significantly higher in the NAR-BFR condition compared to both AR-BFR and no BFR (*p* < 0.05). However, no significant differences were observed between AR-BFR and no BFR (*p* > 0.05). During the second set, significant differences were found between all conditions (*p* < 0.05). In the third and fourth sets, total hemoglobin levels were significantly higher in both BFR conditions (AR-BFR and NAR-BFR) compared to no BFR (*p* < 0.05), with no significant differences observed between the two BFR conditions (*p* > 0.05) (see Table 5 and Figure 2C).

**Table 5:** Average total hemoglobin during each Set (g/dL).

Condition	Set 1	Set 2	Set 3	Set 4
AR-BFR^‡^	12.39 ± 0.37	12.61 ± 0.37*	12.69 ± 0.34*	12.75 ± 0.31*
NAR-BFR^‡^	12.53 ± 0.31	12.73 ± 0.35*	12.81 ± 0.37*	12.87 ± 0.37*
No BFR	12.27 ± 0.32	12.36 ± 0.31*	12.40 ± 0.31*	12.41 ± 0.32

BFR, blood flow restriction; AR-BFR, autoregulated BFR; NAR-BFR, non-autoregulated BFR; g/dL, grams per deciliter.

* Significant time effect.

‡ Significant interaction with no BFR; *α* = 0.05; values expressed as mean ± SD.

Regarding time effects, total hemoglobin levels significantly increased from the first to the second set for both BFR conditions [AR-BFR: 1.68 ± 0.59% [95% CI: 1.39%–1.98%]; NAR-BFR: 1.54 ± 0.98% (95% CI: 1.05%–2.02%)] (*p* < 0.05). Similar increases were observed from the second to the third set [AR-BFR: 0.68 ± 0.39% (95% CI: 0.49%–0.88%); NAR-BFR: 0.63 ± 0.56% (95% CI: 0.35%–0.91%)] and from the third to the fourth set [AR-BFR: 0.46 ± 0.42% (95% CI: 0.25%–0.67%); NAR-BFR: 0.48 ± 0.25% (95% CI: 0.36%–0.60%)] for both BFR conditions (*p* < 0.05). In contrast, for the no BFR condition, total hemoglobin levels significantly increased from the first to the second set [0.80 ± 0.59% (95% CI: 0.51%–1.09%)] and from the second to the third set [0.26 ± 0.32% (95% CI: 0.10% to −0.42%)] (*p* < 0.05), but no significant change was observed from the third to the fourth set [0.14 ± 0.27% (95% CI: 0.01%–0.28%)] (*p* > 0.05).

### Rating of perceived exertion

Our results revealed a significant time × condition interaction for RPE levels, *F*(1,17) = 39.945, *p* < 0.05, *η_p_*^2^ = 0.701. Follow-up analyses comparing RPE levels between conditions during each set showed that RPE was significantly higher in both AR-BFR and NAR-BFR compared to no BFR after each set (*p* < 0.05). However, no significant differences were found between AR-BFR and NAR-BFR (*p* > 0.05) (see Table 6 and Figure 2D).

**Table 6:** Rating of perceived exertion (RPE).

Condition	Set 1	Set 2	Set 3	Set 4
AR-BFR^‡^	3.22 ± 1.22	4.22 ± 1.40*	5.00 ± 1.45*	5.44 ± 1.82
NAR-BFR^‡^	3.56 ± 1.29	4.39 ± 1.79*	5.78 ± 1.83*	6.17 ± 1.98
No BFR	1.79 ± 0.81	2.22 ± 0.94*	2.72 ± 1.27*	3.17 ± 1.50*

BFR, blood flow restriction; AR-BFR, autoregulated BFR; NAR-BFR, non-autoregulated BFR.

* Significant time effect.

‡ Significant interaction with no BFR; *α* = 0.05; values expressed as mean ± SD.

Regarding time effects, perceived exertion significantly increased from the first to the second set for both BFR conditions [AR-BFR: 24.55 ± 14.02% (95% CI: 17.58%–31.52%); NAR-BFR: 15.53 ± 15.12% (95% CI: 8.01%–23.05%)] (*p* < 0.05). Similar increases were observed from the second to the third set [AR-BFR: 15.89 ± 11.68% (95% CI: 10.08%–21.70%); NAR-BFR: 26.19 ± 14.95% (95% CI: 18.75%–33.62%)] (*p* < 0.05). However, no significant changes were observed from the third to the fourth set for either BFR condition [AR-BFR: 3.69 ± 27.67% (95% CI: −10.07% to 17.45%); NAR-BFR: 5.41 ± 11.01% (95% CI: −0.07% to 10.88%)] (*p* > 0.05). In contrast, under the no BFR condition, perceived exertion levels significantly increased across all four sets. Specifically, there was an increase of 18.52 ± 27.94% [95% CI: 4.62%–32.41%] from the first to the second set, 14.26 ± 17.23% [95% CI: 5.59%–22.53%] from the second to the third set, and 11.67 ± 17.39% [95% CI: 3.02%–20.31%] from the third to the fourth set for the no BFR condition (*p* < 0.05).

### Rating of perceived discomfort

Our results revealed a significant time × condition interaction for RPD levels, *F*(1,17) = 6.408, *p* < 0.05, *η_p_*^2^ = 0.274. Follow-up analyses comparing RPD levels between conditions after each set showed that perceived discomfort was significantly higher in both AR-BFR and NAR-BFR compared to no BFR after each set (*p* < 0.05). However, no significant differences were found between AR-BFR and NAR-BFR (*p* > 0.05) (see Table 7 and Figure 2E).

**Table 7:** Rating of perceived discomfort (RPD).

Condition	Set 1	Set 2	Set 3	Set 4
AR-BFR^‡^	3.00 ± 1.19	4.28 ± 1.60*	5.28 ± 1.53*	5.83 ± 1.76*
NAR-BFR^‡^	3.22 ± 1.31	4.50 ± 1.54*	5.33 ± 2.00*	6.00 ± 2.17*
No BFR	1.94 ± 1.16	2.39 ± 1.50	2.72 ± 1.67	3.33 ± 1.78*

BFR, blood flow restriction; AR-BFR, autoregulated BFR; NAR-BFR, non-autoregulated BFR.

* Significant time effect.

‡ Significant interaction with no BFR; α = 0.05; values expressed as mean ± SD.

Regarding time effects, perceived discomfort levels significantly increased from the first to the second set for both BFR conditions [AR-BFR: 28.61 ± 20.39% (95% CI: 18.47%–38.75%); NAR-BFR: 28.96 ± 18.50% (95% CI: 19.75%–38.16%)] (*p* < 0.05). Similar increases were observed from the second to the third set [AR-BFR: 19.31 ± 15.86% (95% CI: 11.42%–27.20%); NAR-BFR: 24.29 ± 12.41% (95% CI: 8.12%–20.47%)], as well as from the third to the fourth set [AR-BFR: 8.52 ± 14.06% (95% CI: 1.52%–15.51%); NAR-BFR: 10.48 ± 12.35% (95% CI: 4.34%–16.63%)] (*p* < 0.05). However, under the no BFR condition, there were no significant changes in perceived discomfort levels from the first to the second set [12.31 ± 19.73% (95% CI: 2.50%–22.13%)] and from the second to the third set [9.91 ± 15.83% (95% CI: 2.04%–17.78%)] (*p* > 0.05). A significant increase in discomfort was observed from the third to the fourth set [22.92 ± 27.36% (95% CI: 9.32%–36.53%)] for no BFR (*p* < 0.05).

## Discussion

The present study investigated the acute physiological and perceptual responses to low-load resistance exercise performed under three conditions: AR-BFR, NAR-BFR, and no BFR. Both BFR conditions led to significantly greater increases in VMO muscle thickness, decreases in oxygen saturation, and rises in total hemoglobin compared to the no BFR condition. Additionally, participants reported significantly higher levels of RPE and RPD under BFR conditions, with no significant differences between AR-BFR and NAR-BFR across any outcome measure. Although autoregulating cuff pressure does not offer a distinct acute advantage over static pressure BFR in terms of physiological or perceptual effects, both BFR approaches produce significant hemodynamic, muscular, and perceptual responses that support hypertrophic adaptations in long-term training regimens.

### Vastus medialis oblique thickness

Our findings showed that both AR-BFR and NAR-BFR significantly (*p* < 0.05) increased VMO thickness compared to the no BFR condition, with no significant differences observed between the two BFR modalities (see Table 3 and Figure 2A). The increased VMO thickness across all conditions can be attributed to transient fluid shifts that occur during exercise. Exercise-induced hyperemia, driven by increases in local muscle metabolism, results in higher capillary pressure and promotes fluid movement into the interstitial and intracellular spaces, which can increase muscle volume (27, 28). This effect is referred to as active or functional hyperemia and is modulated by both rapid and sustained vasodilatory responses to contractile activity (29–31). In the context of BFR, this response is amplified due to restricted venous outflow and partial arterial inflow, which increases the concentration of vasodilatory metabolites and mechanical intramuscular pressure, which can lead to increased plasma filtration and cell volume expansion (32, 33).

Moreover, the mechanical compression of skeletal muscle during rhythmic contractions contributes to a muscle pump effect, which increases the arteriovenous pressure gradient, leading to intramuscular fluid accumulation and transient hypertrophy (34). These dynamic vascular responses, coupled with the elevated extravascular pressure induced by BFR, explain the significantly greater muscle swelling observed in BFR conditions (32, 33). This interpretation is further supported by a recent study (35), which demonstrated that BFR during rhythmic handgrip exercise resulted in significant venous outflow restriction and fluid retention in the working muscles, reinforcing the idea that BFR-induced hemodynamic changes contribute to muscle volume growth and increases in intramuscular pressure. Despite the autoregulated cuff dynamically adjusting pressure during muscle contraction in the AR-BFR condition, no significant difference was found between AR-BFR and NAR-BFR in terms of VMO thickness (see Table 3 and Figure 2A). This may be due to the acute nature of the intervention, where a single session of autoregulated adjustment may not sufficiently alter the magnitude of muscle swelling compared to constant pressure. It is also plausible that both AR-BFR and NAR-BFR achieved a comparable threshold of mechanical and hemodynamic stimulus, inducing similar vascular responses and transient hypertrophy.

Nevertheless, acute muscle swelling may serve as an anabolic signal (36, 37). The resulting cell swelling exerts mechanical tension on the sarcolemma, which has been shown to stimulate protein synthesis and inhibit proteolysis that promote muscle growth (4, 37). This anabolic effect is thought to occur via mechanotransducive pathways that activate the mTORC1 signaling cascade, which is a critical regulator of muscle protein synthesis and hypertrophy (4, 37). The acute increase in VMO thickness observed in this study, although transient, likely reflects the initial phase of this anabolic cascade and represents a meaningful physiological response that may contribute to long-term structural adaptations with repeated training, as observed in longitudinal BFR training studies (38).

Our hypothesis that AR-BFR would cause a smaller increase in VMO thickness due to dynamic pressure regulation, thus reducing occlusive force and the associated cell swelling was not supported. Both AR-BFR and NAR-BFR produced comparable increases in muscle thickness, suggesting that the autoregulation mechanism may not significantly alter muscle volume responses in acute protocols (see Table 3 and Figure 2A). These findings support previous research demonstrating that low-load BFR training can generate sufficient mechanical and metabolic stress to stimulate early hypertrophic responses (39, 40).

### Vastus medialis oblique oxygen saturation

Both AR-BFR and NAR-BFR significantly reduced VMO oxygen saturation (*p* < 0.05) across exercise sets compared to the no BFR condition (see Table 4 and Figure 2B), reiterating the capacity of BFR to induce a localized hypoxic environment within active muscles (1–4). This hypoxia is a hallmark of BFR-induced metabolic stress, leading to greater reliance on anaerobic metabolism, activation of fast-twitch muscle fibers, and the stimulation of hypertrophic pathways like mTORC1 (4, 37, 41). The decrease in oxygen saturation corresponds with increased oxygen extraction during exercise, where active muscles utilize up to 70%–80% of the oxygen delivered, compared to 20%–40% at rest (42). This significant increase in oxygen extraction results from heightened metabolic demand, an increased arteriovenous oxygen difference, and lower perivascular partial pressure of oxygen levels in active muscle (42).

Under BFR conditions, the restricted arterial inflow and occluded venous outflow intensify intramuscular hypoxia (1–4). This prolongs the accumulation of metabolites such as adenosine, potassium ions, and lactate, while also increasing the deformation of vascular walls due to rhythmic contractions (42–45). These changes enhance vasodilatory responses and oxygen unloading from hemoglobin, which is potentiated by increased hydrogen ion and carbon dioxide levels that shift the oxyhemoglobin dissociation curve (42). Additionally, the muscle pump mechanism and post-contraction hyperemia increase the arteriovenous pressure gradient, enhancing oxygen extraction and facilitating tissue perfusion during rest intervals (34, 46).

Interestingly, although the AR-BFR condition demonstrated relatively higher oxygen saturation during the first two sets compared to NAR-BFR, this pattern reversed during the final two sets, with AR-BFR showing the lowest saturation levels (see Table 4 and Figure 2B). While this shift was not significant, it suggests that autoregulation may initially buffer sharp oxygen desaturation by adjusting cuff pressure during muscle contraction to preserve perfusion in the early phase. However, repeated contractions likely overwhelmed the autoregulatory mechanism, resulting in comparable desaturation compared to static pressure. Our findings highlight the overall metabolic demand of BFR and highlight the dynamic interaction between mechanical occlusion and systemic cardiovascular responses.

From a physiological standpoint, acute decreases in oxygen saturation are expected during high-repetition, low-load BFR protocols due to high metabolic turnover and the activation of multiple vasodilatory pathways (42–45). These include ATP and adenosine release, nitric oxide and prostaglandin signaling, and shear stress-induced vasodilation, all of which are amplified under hypoxic and ischemic conditions commonly seen in BFR (42–45). Red blood cell deformation and hypoxia-induced ATP release further contribute to local vasodilation and blood flow regulation, which support oxygen delivery despite reduced saturation (47, 48).

Our hypothesis anticipated that AR-BFR would result in attenuated decreases in VMO oxygen saturation due to its pressure-adjusting settings. Although not significant (*p* > 0.05), this pattern was observed in the initial exercise sets but was not consistently maintained throughout the protocol. Our findings suggest that the autoregulation may be time- or intensity-dependent and may not significantly mitigate oxygen desaturation over longer or more fatiguing protocols. Furthermore, our results highlight the complex and multifaceted regulation of skeletal muscle oxygenation during BFR exercise, as well as the roles of both local and systemic mechanisms in matching oxygen delivery to demand. Despite lower oxygen saturation values, particularly in BFR conditions, these acute reductions may serve as important signals for muscle adaptation, contributing to both hypertrophic (49, 50) and endurance-related gains (51, 52) with repeated exposure.

### Vastus medialis oblique total hemoglobin

Total hemoglobin (THb) levels significantly (*p* < 0.05) increased in both BFR conditions compared to the no BFR condition, particularly during the later sets of the exercise protocol (see Table 5 and Figure 2C). These elevations reflect enhanced blood pooling and hemoconcentration distal to the cuff, which is consistent with the physiological responses of skeletal muscle during rhythmic contractions and vascular occlusion (52, 53). The mechanical effects of muscle contractions, including increased extravascular pressure, compress the veins and partially occlude arterial inflow, which increase the arteriovenous pressure gradient and elevate THb concentrations in the muscle (29, 54, 55).

The increased THb values can also be attributed to exercise-induced hyperemia, which augments microvascular perfusion, expands functional capillary density, and increases microvessel hematocrit (42). This effect enhances the surface area for exchange and improves oxygen and nutrient delivery during muscular activity (42). In BFR conditions, the restriction of venous return further intensifies these hemodynamic effects, contributing to increased microvascular pressure and fluid filtration that are closely tied to cell swelling and anabolic signaling cascades (32, 33).

Notably, the NAR-BFR condition produced the highest THb levels across most time points, likely due to the absence of dynamic cuff modulation and the static occlusive pressure throughout the exercise bout (see Table 5 and Figure 2C). This may prolong venous stasis and increase local blood volume accumulation (32). In contrast, the AR-BFR condition, while still substantially increasing THb levels, modulated cuff pressure in response to muscle contractions (12, 56). This likely facilitated some degree of transient perfusion recovery during the contraction-relaxation cycles, potentially tempering the magnitude of hemoconcentration without eliminating its physiological benefits.

Our hypothesis anticipated that AR-BFR would induce a lower THb response due to real-time cuff pressure adjustments, which was partially supported during the early sets. By the third and fourth sets, however, both AR-BFR and NAR-BFR conditions produced similarly elevated THb levels (see Table 5 and Figure 2C). This finding suggests that autoregulatory pressure control may buffer hemodynamic stress transiently but does not prevent cumulative hemodynamic responses under repeated contractions. These findings further reinforce that both autoregulated and non-autoregulated BFR settings elicit comparable vascular stress and metabolic conditions necessary to drive acute adaptation. The THb findings demonstrate the critical role of hemodynamic shifts and vascular responses during BFR conditions compared to no BFR. Furthermore, both AR-BFR and NAR-BFR protocols effectively increased THb levels in the VMO, contributing to a cellular and metabolic environment favorable for muscle growth (4, 37, 41).

### Perceptual responses

Our results showed that both AR-BFR and NAR-BFR significantly (*p* < 0.05) increased subjective ratings of exertion (RPE, see Table 6 and Figure 2D) and discomfort (RPD, see Table 7 and Figure 2E) compared to the no BFR condition. These perceptual findings align with previous research demonstrating that BFR induces substantial metabolic and hemodynamic stress, which can amplify sensory feedback and central fatigue, even during low-load resistance exercise (56, 57). The elevated RPE likely reflects the response to ischemia (45, 58), metabolite accumulation (43, 45), and increased motor unit recruitment (41) required to maintain force output under hypoxic conditions. Simultaneously, the increased RPD in the BFR conditions suggests that restricted blood flow produces greater nociceptive input due to sustained venous pooling and rising interstitial pressure (32, 33). Additionally, BFR causes localized metabolic stress and mechanical forces, which can stimulate pain-sensitive free nerve endings, particularly when the muscle contracts repeatedly under hypoxic conditions (59, 60).

The total number of 75 repetitions performed per set (1 × 30, 3 × 15) is also a key contributor to the elevated perceptual burden. This protocol induces progressive metabolic stress and a substantial increase in local blood volume and hemoconcentration, which enhances both cell swelling and metabolite trapping, two mechanisms thought to stimulate mTORC1 signaling and muscle growth (4, 37). However, these same mechanisms also increase the perceived effort and discomfort, which may potentially pose a barrier to long-term adherence. Perceptual demand has been identified as a critical determinant of training adherence and program compliance, particularly in clinical or older adult populations (61). Despite the higher RPE and RPD observed with BFR, it is essential to note that BFR uniquely increases metabolic stress while minimizing mechanical load on the joints (1, 2).

Although all participants were able to complete the full 75 repetitions (1 × 30, 3 × 15) across all conditions, the significantly higher RPE and RPD levels observed in the BFR conditions suggest that BFR training is more physically demanding compared to no BFR. This heightened perceptual discomfort likely reflects the greater metabolic stress induced by BFR, which may play a role in enhancing hypertrophic adaptations (39, 40). While the BFR protocols were more challenging, they provide an effective strategy for promoting muscle growth under low mechanical loads, especially in populations where high-load training may not be feasible.

## Limitations

Several limitations should be considered when interpreting the findings of this study. First, the sample consisted of healthy, young, and recreationally active adults, which may limit the generalizability of the results to clinical populations such as individuals with patellofemoral pain syndrome or knee osteoarthritis. While this homogeneous sample was suitable for examining acute physiological responses, future studies should include clinical populations to better understand the therapeutic relevance of BFR in individuals with pain, functional limitations, or comorbidities. Second, this study investigated only the acute effects of a single exercise session. Although acute responses did not differ, a long-term study could explore the effects of a progressively and consistently performed BFR exercise program, potentially revealing changes in perceptual responses and muscle adaptations. Third, this study used a single VMO-targeted exercise (single-leg wall squat), which may not fully represent the range of motor unit recruitment or load distribution seen in multi-joint functional training. Moreover, since we did not assess 1RM strength, it is likely that participants were exercising at different relative loads. However, the within-subjects design employed reduces the magnitude of impact of individual participants as they all serve as their own controls and are included within all three comparisons. Future research should investigate various types of exercise, incorporate relative loads where relevant, extend intervention durations, and include functional assessments such as gait, strength, and pain levels to expand on these preliminary findings. Furthermore, this study used a wide and rigid nylon cuff for BFR training with a well-studied autoregulatory function with validated maintenance of interface pressures during rest and exercise. Since previous research has shown differences in physiological responses based on cuff width and material (62, 63), as well as showing difference capacities for cuffs to maintain interface pressure (24), future studies should examine how various cuff features, such as width, elasticity, and material, interact with the autoregulatory feature of other commonly used BFR cuff systems to induce physiological responses.

## Conclusions

In conclusion, both BFR modalities (AR-BFR and NAR-BFR) resulted in significantly greater acute increases in VMO muscle thickness, elevated total hemoglobin concentrations, and greater reductions in muscle oxygen saturation compared to the no BFR condition. These acute responses reflect the distinct physiological environment created by BFR training, characterized by partially occluded arterial inflow, restricted venous outflow, increased muscle cell swelling, and reduced oxygen availability within the muscle. This hypoxic environment has been shown to activate key anabolic pathways that promote protein synthesis and muscle hypertrophy even in the absence of high mechanical loading. Despite differences in cuff pressure regulation, AR-BFR and NAR-BFR elicited comparable hemodynamic and perceptual responses, suggesting that both methods can be equally effective in the short term for inducing acute muscular adaptations. Importantly, these effects were achieved using low-load exercise, which may reduce joint compressive forces and mechanical strain. These factors are particularly relevant to populations with anterior knee pain, including those with patellofemoral pain syndrome or knee osteoarthritis. Therefore, BFR represents a clinically relevant strategy to promote quadriceps, and specifically VMO, hypertrophy in individuals who cannot tolerate traditional high-load resistance training. Integrating BFR into rehabilitation or prehabilitation programs may enhance patellar stability, reduce pain, and improve functional outcomes in patients with compromised joint integrity or load sensitivity. Future research is warranted to examine the long-term training adaptations of autoregulated vs. non-autoregulated BFR, particularly in clinical populations such as individuals with patellofemoral pain syndrome or knee osteoarthritis.

## Data Availability

The raw data supporting the conclusions of this article will be made available by the authors, without undue reservation.
